# Modular Molecular Weaponry Plays a Key Role in Competition Within an Environmental *Vibrio cholerae* Population

**DOI:** 10.3389/fmicb.2021.671092

**Published:** 2021-05-21

**Authors:** Nora A. S. Hussain, Paul C. Kirchberger, Rebecca J. Case, Yann F. Boucher

**Affiliations:** ^1^Department of Biological Sciences, University of Alberta, Edmonton, AB, Canada; ^2^Department of Integrative Biology, University of Texas at Austin, Austin, TX, United States; ^3^Singapore Centre for Environmental Life Sciences Engineering (SCELSE), Nanyang Technological University, Singapore, Singapore; ^4^School of Biological Sciences, Nanyang Technological University, Singapore, Singapore; ^5^Saw Swee Hock School of Public Health, National University of Singapore, Singapore, Singapore

**Keywords:** *Vibrio cholerae*, type VI secretion system, lateral gene transfer, intraspecific conflict, bacterial population structure

## Abstract

The type VI secretion system (T6SS) operons of *Vibrio cholerae* contain extraordinarily diverse arrays of toxic effector and cognate immunity genes, which are thought to play an important role in the environmental lifestyle and adaptation of this human pathogen. Through the T6SS, proteinaceous “spears” tipped with antibacterial effectors are injected into adjacent cells, killing those not possessing immunity proteins to these effectors. Here, we investigate the T6SS-mediated dynamics of bacterial competition within a single environmental population of *V. cholerae*. We show that numerous members of a North American *V. cholerae* population possess strain-specific repertoires of cytotoxic T6SS effector and immunity genes. Using pairwise competition assays, we demonstrate that the vast majority of T6SS-mediated duels end in stalemates between strains with different T6SS repertoires. However, horizontally acquired effector and immunity genes can significantly alter the outcome of these competitions. Frequently observed horizontal gene transfer events can both increase or reduce competition between distantly related strains by homogenizing or diversifying the T6SS repertoire. Our results also suggest temperature-dependent outcomes in T6SS competition, with environmental isolates faring better against a pathogenic strain under native conditions than under those resembling a host-associated environment. Taken altogether, these interactions produce density-dependent fitness effects and a constant T6SS-mediated arms race in individual *V. cholerae* populations, which could ultimately preserve intraspecies diversity. Since T6SSs are widespread, we expect within-population diversity in T6SS repertoires and the resulting competitive dynamics to be a common theme in bacterial species harboring this machinery.

## Introduction

Environmental populations of *Vibrio cholerae*, the etiological agent of cholera, are composed of different toxigenic and non-toxigenic lineages living together in brackish water habitats (Pretzer et al., 2017; Mavian et al., 2020). Among their competitive arsenal, shared with ∼25% of all Gram-negative bacteria (Bingle et al., 2008), is the type VI secretion system (T6SS). T6SS structural genes are spread over three loci termed aux-1, aux-2, and large cluster in the *V. cholerae* genome (Unterweger et al., 2014). Components of these loci form the T6SS apparatus, which has evolved from a bacteriophage tail spike (Pukatzki et al., 2007; Bock et al., 2017; Nguyen et al., 2017). It consists of a hollow tube tipped with a membrane-puncturing protein spear, surrounded by an outer sheath (Leiman et al., 2009; Zoued et al., 2014). Contraction of the outer sheath propels the tip of the spear and interior tube into adjacent cells (including cells of the same species or even clonal lineage), injecting a combination of potentially lethal effector proteins in a contact-dependent manner (Unterweger et al., 2014). Effectors confer a variety of cytotoxic abilities and are each generally encoded upstream of a specific immunity gene, forming effector-immunity (E-I) modules. Survival of T6SS-mediated attacks depends on cells possessing the correct combination of immunity proteins to neutralize incoming effectors (Ho et al., 2014; Smith et al., 2020). As such, even closely related cells with different immunity proteins are killed through T6SS-mediated antagonism (Unterweger et al., 2014). In terms of E-I module content, the aux-1, aux-2, and large clusters of *V. cholerae* are polymorphic, each capable of encoding a large variety of different E-I modules. Additionally, three monomorphic T6SS loci (encoding only a single E-I module) have also been identified in *V. cholerae* and designated as aux-3, aux-4, and aux-5 (Altindis et al., 2015; Labbate et al., 2016; Crisan et al., 2019). Given the large number of E-I modules, strains of *V. cholerae* could theoretically display millions of different combinations, and indeed the observed strain level diversity in T6SS module combinations is vast (Kirchberger et al., 2017).

This degree of variation in E-I profiles is mainly attributable to horizontal gene transfer (HGT) (Kirchberger et al., 2017), which, in *V. cholerae*, is tightly linked with T6SS activity (Borgeaud et al., 2015; Thomas et al., 2017; Matthey et al., 2019). The acquisition and replacement of effector and/or immunity genes is orchestrated through various recombination mechanisms, namely, homologous recombination and homology-facilitated illegitimate recombination for polymorphic loci (Cooper et al., 2017; Kirchberger et al., 2017; Thomas et al., 2017) and site-specific recombination in the case of monomorphic loci (Miyata et al., 2013a; Labbate et al., 2016; Santoriello et al., 2020). Ancestral immunity genes can be entirely replaced or retained with the addition of new E-I modules within a locus (Kirchberger et al., 2017). Retention of immunity modules during recombination events, replacing their cognate effector, can lead to the accumulation of multiple orphan immunity genes at a single locus (Kirchberger et al., 2017). These orphan immunity genes are hypothesized to provide a fitness advantage similar to acquired interbacterial defense islands, protecting strains from additional T6SS attacks (Ross et al., 2019).

Despite the large E-I module assortment within *V. cholerae*, almost all members of the pandemic generating (PG) lineage responsible for cholera (Islam et al., 2017) have retained one specific E-I module combination (Unterweger et al., 2014; Kirchberger et al., 2017). Due to their nature as the causative agent of cholera, their T6SS has been the most closely studied (Ma et al., 2009; MacIntyre et al., 2010; Unterweger et al., 2012; Miyata et al., 2013b; Kostiuk et al., 2017; Zhao et al., 2018; Fast et al., 2020). The T6SS is under strict regulation in PG strains, with activation modulated through numerous variables, including temperature, osmolarity, cell density, as well as mucin, indole, and bile salts present in the human digestive tract (Bernardy et al., 2016; Joshi et al., 2017; Kostiuk et al., 2017). This contrasts with the constitutively active T6SS in most non-toxigenic environmental strains thriving in temperate and tropical aquatic environments (Unterweger et al., 2012; Bernardy et al., 2016; Drebes Dorr and Blokesch, 2020). In competition with environmental strains, the specific E-I module combination of PG *V. cholerae* has been hypothesized to be superior to that of other strains, making PG *V. cholerae* shed by patients successful competitors against environmental bacteria (Miyata et al., 2010; Unterweger et al., 2014; Kostiuk et al., 2017).

However, while the vast majority of research is focused on pathogenic *V. cholerae*, most members of this species are (I) not host-associated and (II) not virulent. The apparent superiority of a specific E-I module combination is thus not consistent with the HGT-mediated diversity in E-I modules observed in environmental vibrios (Dar et al., 2018; Drebes Dorr and Blokesch, 2020) and other bacteria (Wexler et al., 2016; Steele et al., 2017; Verster et al., 2017; Lewis et al., 2019; Wu et al., 2019). This diversity is expected to play an important role in establishing a niche in unoccupied surfaces (Speare et al., 2018) (primary colonization), defending an existing biofilm from invaders (Hecht et al., 2016), and initiating attacks on an existing microbial community (Vacheron et al., 2019) (secondary colonization). As the majority of *V. cholerae* has been shown to be associated with particles in the environment (Kirchberger et al., 2020), these high-density conditions allow effective T6SS competition through limited physical distancing between cells and serve as ideal microcosms for contact-dependent competition to occur.

Yet, to this date, the impact of the T6SS in shaping natural bacterial populations (i.e., groups of co-occurring and co-evolved strains of bacteria of a single species in a single location) has not been elucidated. In this study, we investigate the competitive dynamics between primary colonizers on a solid surface created by the diversity of T6SS E-I modules. Through pairwise competition of the 14 most prevalent strains in a coastal *V. cholerae* population, almost all of which contain unique E-I modules, this study uncovers a network of interaction that is shaped by environmental factors, population structure and frequent HGT.

## Results

### Almost all Lineages in a *Vibrio cholerae* Population Possess Mutually Incompatible T6SS E-I Module Compositions

**FIGURE 1:**
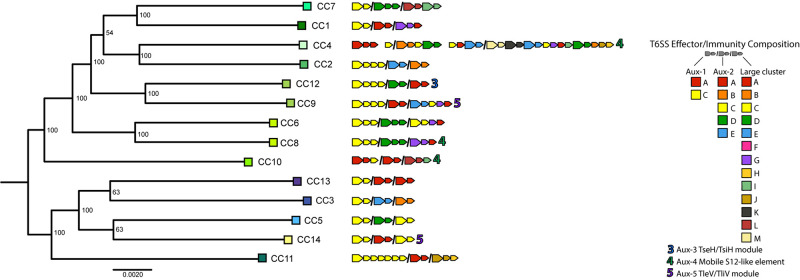
Type VI secretion system effector and immunity genes in a *Vibrio cholerae* population. Representative strains of clonal complexes in the *V. cholerae* population of Oyster Pond (MA, United States) were typed for their T6SS gene complement. Large arrows next to strain names indicate T6SS effectors, small arrows indicate immunity genes, and color of arrows represent different effector and immunity protein coding gene families as defined by >30% shared amino acid identity. Auxiliary clusters 1 and 2 and the large cluster genes are separated by slashes; spaces within clusters denote interruptions in canonical locus structure. Note that similarly named/colored families denote different families in different clusters. Numbers indicate auxiliary T6SS loci. Strain phylogeny was constructed from a 2,948,969-bp whole genome alignment using the GTR + GAMMA model implemented in RAxML (Stamatakis, 2014). Statistical branch support was obtained from 100 bootstrap pseudoreplicates. Tree was rooted at the midpoint.

To understand T6SS-mediated competitive dynamics in *V. cholerae* populations, we analyzed the structure of T6SS loci in 14 dominant strains from an extensively sampled coastal population in the eastern United States (Kirchberger et al., 2016, 2020). Clonal complexes (CCs) are groups of very closely related strains, as initially determined by multi-locus sequence typing, with approximately 40,000–50,000 single-nucleotide polymorphisms (less than 99% Average Nucleotide Identity) between CCs. For typing the T6SS loci of strains representing these CCs, we followed previously developed typing schemes (Unterweger et al., 2014; Kirchberger et al., 2017), denoting effector and cognate immunity gene families in the T6SS-associated large cluster and aux-1 and aux-2 with capital letters (Figure 1). In the case of aux-1 and aux-2, this scheme describes auxiliary toxins loaded onto the T6SS spear by adapter proteins, and in the case of the large locus, variable C-termini of the spear-forming VgrG protein itself (Unterweger et al., 2014; Kirchberger et al., 2017). Additional monomorphic loci aux-3, aux-4, and aux-5 were also characterized based on previous studies (Altindis et al., 2015; Labbate et al., 2016; Crisan et al., 2019). In accordance with what is observed in *V. cholerae* on a global scale, almost every member of this individual population encodes a unique combination of T6SS E-I modules (Figure 1), with only three possessing a counterpart in a previously described global *V. cholerae* dataset (Kirchberger et al., 2017). E-I structures ranged from simple one effector–one immunity gene pairings at each of the three main loci (for example CC3 or CC5) to a complex array containing not only a complete E-I-pair but also three truncated effectors and their cognate immunity genes, and seven additional orphan immunity genes in the large locus of CC4. Overall, out of the 19 effector and cognate immunity gene families previously observed in the *V. cholerae* pangenome, only three effectors (C in aux-2, and F and H in the large cluster) and only one cognate immunity gene (F in the large cluster) are absent in the population. A previously undescribed putative E-I module type was also observed in the large cluster of CC4, consisting of a unique C-terminal region and a cognate immunity gene (WP_141239147), here termed M-type in accordance with the alphabetical naming scheme. As previous experimental results show that strains possessing different E-I module combinations are capable of mutual killing, and assuming that orphan immunity genes are capable of detoxifying cognate effectors, the vast majority of strain combinations within this population should not be able to coexist in close contact without mutual T6SS-mediated killing (i.e., they should be incompatible) (Supplementary Figure 1).

### T6SS Competition Between Aquatic *Vibrio cholerae* Strains Is Constitutive Yet Temperature Dependent

**FIGURE 2:**
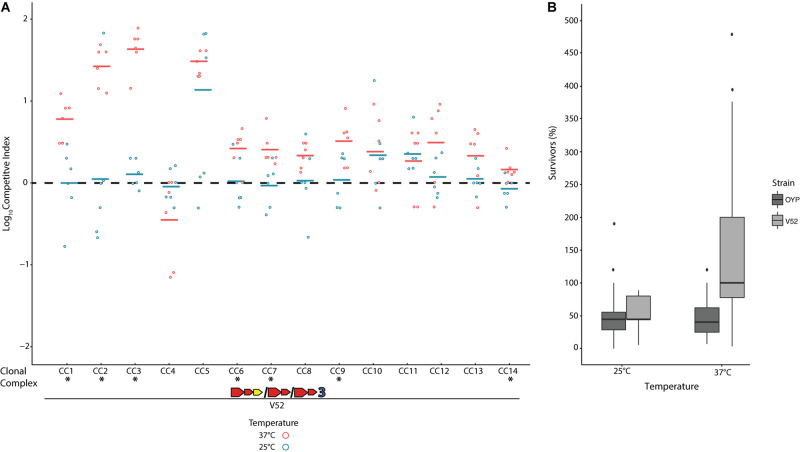
Competition of a toxigenic Pandemic Generating lineage *Vibrio cholerae* strain against non-toxigenic natural isolates at environmental and human host temperatures. The clinical *V. cholerae* strain V52 (Zinnaka and Carpenter, 1972) was competed on agar plates against environmental *V. cholerae* isolates from Oyster Pond (MA, United States) in equal concentration at either 25°C or 37°C over 4 h. **(A)** Dots indicate the log competitive index (CI) for each replicate (*N* = 6) at 37°C (red) and 25°C (blue), with the average shown as a bar on the *y*-axis. A positive log CI indicates a favorable outcome toward V52, and a negative CI denotes environmental strains as the winner. Asterisks (*) indicate statistically significant differences in mean CI values between 25°C and 37°C competitions from two-tailed, unpaired *t* tests (*p* < 0.05). **(B)** Percentage of surviving cells compared to the original input for V52 and all environmental strains after 4 h of competition at 25°C and 37°C. Box-and-whiskers plots show median, 25th and 75th percentiles (upper and lower hinges), and 1.5 interquartile range (whiskers). Outliers are shown as individual dots.

When pitted against *Escherichia coli* K12 through co-incubation on agar plates at 37°C, all *V. cholerae* strains showed the ability to outcompete this bacterium, which does not encode a T6SS (Supplementary Figure 2). This competitive outcome was due to the death of over half of all *E. coli* cells due to direct physical contact with *V. cholerae* (Supplementary Figure 2B), and no reduction in *E. coli* cell numbers was observed from exposure to *V. cholerae* supernatants alone or in controls with *E. coli* only. The T6SS of these isolates is thus constitutively active under the conditions of these competition assays, as has previously been shown for environmental *V. cholerae* (Bernardy et al., 2016; Drebes Dorr and Blokesch, 2020). Temperature has been reported to affect T6SS expression in some strains (Ishikawa et al., 2012; Townsley et al., 2016) and could likely affect the outcome of competition. To test this hypothesis, we competed all environmental strains against *V. cholerae* strain V52, a toxigenic PG lineage isolate with constitutively active T6SS expression and superior competitive abilities compared to a number of environmental strains (MacIntyre et al., 2010; Miyata et al., 2013b; Unterweger et al., 2014; Kostiuk et al., 2017). The V52 E-I module combination (AAA) is not found in any Oyster Pond strains and therefore should be incompatible with all of them. When co-incubated at 37°C, V52 clearly outcompeted nine out of 14 strains (CC1–CC3, CC5–CC9, and CC14, *p* > 0.05, Figure 2). Only CC4, a strain displaying unusually gene-rich T6SS clusters (see Figure 1), appeared to show a degree of (non-significant) competitive ability against V52 at 37°C. However, this temperature is never observed in Oyster Pond, which at most reaches 28°C (Kirchberger et al., 2020). At 25°C, the average summer temperature of Oyster Pond (Kirchberger et al., 2020), we observed a general trend toward increased competitive ability for environmental strains. Seven out of 14 strains fared significantly better against V52 at this temperature, with only CC11 showing a degree of reduced competitive ability (Figure 2). Overall, only two out of 14 strains were outcompeted by V52 at 25°C (CC5 and CC11). This altered outcome of competitions at lower temperature was likely not the result of down-regulation of T6SS activity, as cytotoxicity was not reduced. Indeed, at both 37°C and 25°C, about half the cells of environmental competitors were killed by V52 (Figure 2B), indicating unaltered T6SS activity. In contrast, the number of surviving V52 cells changed from 200% of the original input (indicating growth) at 37°C to lower than 100% at 25°C. Therefore, either a decrease in relative growth rate for V52, increased T6SS-mediated competitive ability of environmental strains, or a combination of both, in response to altered temperature, resulted in much more even matchup between the competitors.

### Genetically Divergent Strains With Compatible T6SS Experience Limited Competition

**FIGURE 3:**
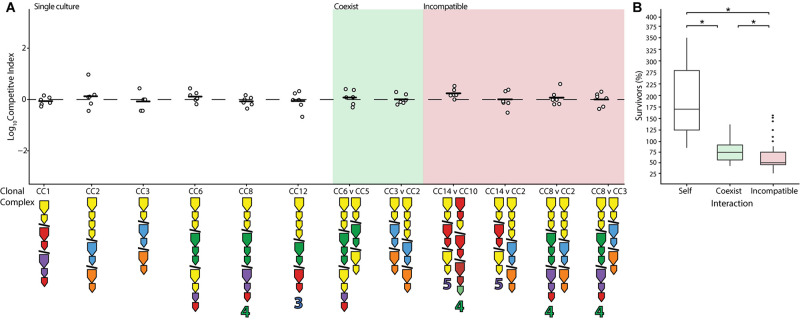
Reduction of competition between strains with identical effector-immunity module composition. Identical (white), compatible (green), and select incompatible (red) strains were competed on agar plates at equal concentrations at 25°C for 4 h. **(A)** Dots indicate the log competitive index for each replicate (*N* = 6), with the average shown as a bar on the *y*-axis. E-I module composition is indicated below strain names in accordance with Figure 1. **(B)** Percentage of surviving cells compared to the original input of identical (white), compatible (green), and incompatible (red) strains with non-significant competitive outcomes (excluding instances of one strain outcompeting the other). Box-and-whiskers plots show median, 25th and 75th percentiles (upper and lower hinges), and 1.5 interquartile range (whiskers). Outliers are shown as individual dots. Asterisks (*) indicate statistically significantly different means between each group (unpaired *t* test *p* < 0.05).

Previous work has shown that while closely related strains of *V. cholerae* (members of the PG lineage) engage each other using their T6SS, this interaction does not result in damage, presumably because their identical E-I modules neutralize one another (Unterweger et al., 2014). Assuming that identity in E-I module composition solely determines whether strains coexist (rather than other genetic factors), it can be predicted that almost all combinations of *V. cholerae* isolated from the Oyster Pond population should be incompatible (Supplementary Figure 1). In two instances (CC5 and CC6 as well as CC2 and CC3), phylogenetically divergent strains possess similar E-I module composition, differing only in orphan immunity genes, and could therefore potentially coexist (Figure 1 and Supplementary Figure 1). To test this prediction, pairwise competition assays were performed using all possible combinations of compatible strains. In accordance with the findings that temperature significantly influences the outcome of competition, all assays were performed at 25°C to emulate environmental conditions. Indeed, both specific predictions of coexistence resulted in the log of competitive indices (CIs) being close to zero, an outcome identical to competitions of strains with themselves (Figure 3A). The results in Figure 3 further indicate that rifampicin resistance did not affect competition, as self-crosses performed as expected in wild-type (WT) outcomes. However, while competition of a strain with itself resulted in near doubling of cell numbers, competition of different strains with identical E-I modules yielded reduced numbers of both cell types (Figure 3B). Nonetheless, the number of cells surviving competition with strains bearing compatible E-I module composition was still significantly higher (*p* < 0.05) than that of incompatible strains, with (on average) 75% of cells of each lineage surviving instead of only 50% (Figure 3B). Incomplete detoxification due to suboptimal binding of effectors by divergent immunity proteins could be the reason for the remaining degree of competition (Zhang et al., 2013; Alteri et al., 2017). Indeed, the aux-1 immunity proteins of CC2 and CC3 (68.5–72.8% average amino acid identity) and the aux-2 immunity proteins of CC5 and CC6 (82.4% identity) display a considerable degree of divergence. Overall, however, it is clear that even distantly related strains show reduced levels of competition when possessing the same E-I module types.

### Horizontally Transferred E-I Modules Reduce Competition Between Distantly Related Strains

Since E-I modules of the same type are patchily distributed across the diversity of the *V. cholerae* species, it is likely that they have spread through HGT events. In particular, compatible strains CC2 and CC3’s closest relatives (CC4 and CC13, respectively) display different E-I module compositions (Figure 1). Their T6SS compatibility is therefore not always inherited from common ancestors. However, it is unclear whether these HGT events happened in the distant past, with compatible strains acquiring modules elsewhere and migrating into the extant population independently, or whether strains acquired them from each other in the currently existing population, homogenizing E-I module content. To investigate these possibilities, homologous effector and immunity genes from all Oyster Pond and reference strains with sequenced genomes were aligned and their phylogeny was reconstructed. Recent, in-population HGT events should be apparent by identical or nearly identical E-I module sequences in divergent but compatible strains, resulting in monophyletic clades consisting only of Oyster Pond isolates. In the case of aux-1, which displays the most sequence variation and least E-I module type diversity, recent in-population HGT is apparent between CC3 and CC7, which present almost identical C-type effector and immunity genes but incompatible E-I modules in other loci (Supplementary Figure 2A). In contrast, genes for compatible strain pairs CC2–CC3 and CC5–CC6 appear to have diverged long ago or have been acquired independently, as evidenced by their phylogenetic distance. In the aux-2 loci, D-type effector and immunity genes for incompatible strain pairs CC4–CC12 as well as CC6–CC7 cluster together and apart from the alleles of all other strains, which is evidence of recent in-population HGT (Supplementary Figure 2B). In the large cluster, the B-type E-I pair of compatible strains CC2 and CC3 forms a well-supported, monophyletic clade distinct from all other Oyster Pond or reference E-I modules of the same type (Supplementary Figure 2C). Immunity genes from both strains as well as the 3′-half of their effectors are identical in sequence, which is strongly suggestive of a recent recombination event.

### Orphan Immunity Genes in the Large T6SS Cluster Can Confer a Competitive Advantage

**FIGURE 4:**
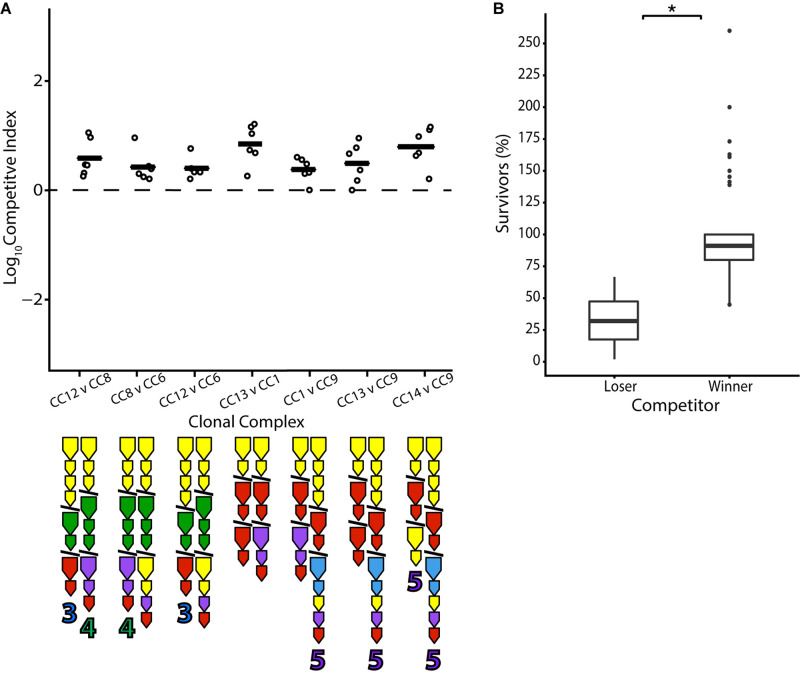
Orphan immunity genes confer an advantage in pairwise competition. Strains differing only in orphan immunity gene composition were competed in equal concentration at 25°C over 4 h. **(A)** Dots indicate the log competitive index for each replicate (*N* = 6), with the average shown as a bar on the *y*-axis, with a positive CI indicating a favorable competitive outcome for the second strain in the competition. All CI values were statistically significant based on a two-tailed, one-sample Student’s *t* test (*p* < 0.05). E-I module composition is indicated below strain names. **(B)** Percentage of surviving cells compared to the original input of losing and winning strains in individual competitions. Box-and-whiskers plots show median, 25th and 75th percentiles (upper and lower hinges), and 1.5 interquartile range (whiskers). Outliers are shown as individual dots. Asterisk indicates statistically significantly different means between the anticipated losing and winning groups (unpaired *t* test *p* < 0.05).

T6SS immunity genes without matching effectors in a genome have been termed “orphans” (Russell et al., 2012). Such orphans are present in a large number of *V. cholerae* strains in the population and are theoretically capable of conferring protection to the effectors of other bacteria. From the E-I profiles present in this set of isolates, it is predicted that in around a fifth of all possible unique combinations (18/91), a strain would be immune to all effectors of its competitor but not vice versa (Supplementary Figure 1). Indeed, with two exceptions, pairwise competition resulted in a positive outcome for the strain possessing orphan immunity genes compatible with their opponent’s effector from the large cluster. For example, CC12 is outcompeted by CC8 (Figure 4), which is protected from CC12’s A-type effector in the large locus by an orphan A-type immunity gene but possesses a G-type effector that CC12 is not protected against. However, both CC8 and CC12 are outcompeted by CC6, which possesses orphan A and G-type immunity genes and a C-type effector. Notably, aux-3 of CC12 and aux-4 of CC8—which are mobile genetic elements encoding additional, unrelated E-I modules (Altindis et al., 2015; Labbate et al., 2016; Crisan et al., 2019)—did not appear to sway the outcome of the competition (Figure 4). Similarly, CC13 is outcompeted by CC1, and they are both outcompeted by CC9, whose three orphan immunity genes are predicted to protect against their effectors (Figure 4). However, not all results were clear-cut: the non-Oyster-Pond strain V52, which should be immune to all of CC13’s effectors, did not significantly outcompete it (Figure 2). The protective orphan immunity protein of V52 displays only 80% amino acid identity from the immunity protein that protects CC13 against its own effector, raising the possibility that the protection conferred to V52 is suboptimal.

CC4, a strain that possesses orphan immunity genes to 18 out of all 20 known putative T6SS effectors in *V. cholerae*, largely defied predictions. CC4’s immunity genes theoretically protect it from all effectors in the population with the exception of the large locus L-effectors of CC7 and CC10. While CC4 outcompetes four strains according to expectations, it is equally matched with five others and is outcompeted by two other strains. However, the structure of the T6SS loci in CC4 is more complex than in other strains. In aux-1, two A-type immunity genes are followed by a ∼2300-bp region encoding four hypothetical genes, followed by a C-type immunity gene. Failure to express this last gene due to potential exclusion from the normal aux-1 promoter activity would leave CC4 unprotected from the C-type immunity genes of all but one other strain in the population. Similarly, the aux-2 locus is split into two, with only the region containing B- and D-type E-I module and one A-type orphan immunity gene appearing intact and part of the standard operon. The second part containing C- and A-type orphan immunity genes and an E-type E-I module is located downstream of an integrase as well as several genes encoding proteins of unknown function, in a reading frame opposite to that of the other T6SS associated genes. As the region downstream of the T6SS aux-2 locus is conserved among other all strains, it appears that this is an insertion interrupting this region, potentially interfering with expression of these genes. As such, it is clear that complicating factors, such as uncertainty regarding expression in atypical T6SS-associated gene regions and divergence between effector and immunity proteins of the same type, can weaken the overall predictability of competition. Nonetheless, our results show that the presence of additional immunity genes could provide a considerable advantage to *V. cholerae* strains and is one of the most important factors in determining outcomes of intraspecies competition.

### *Vibrio cholerae* Populations Form Dynamic, Competitive Interaction Networks

**FIGURE 5:**
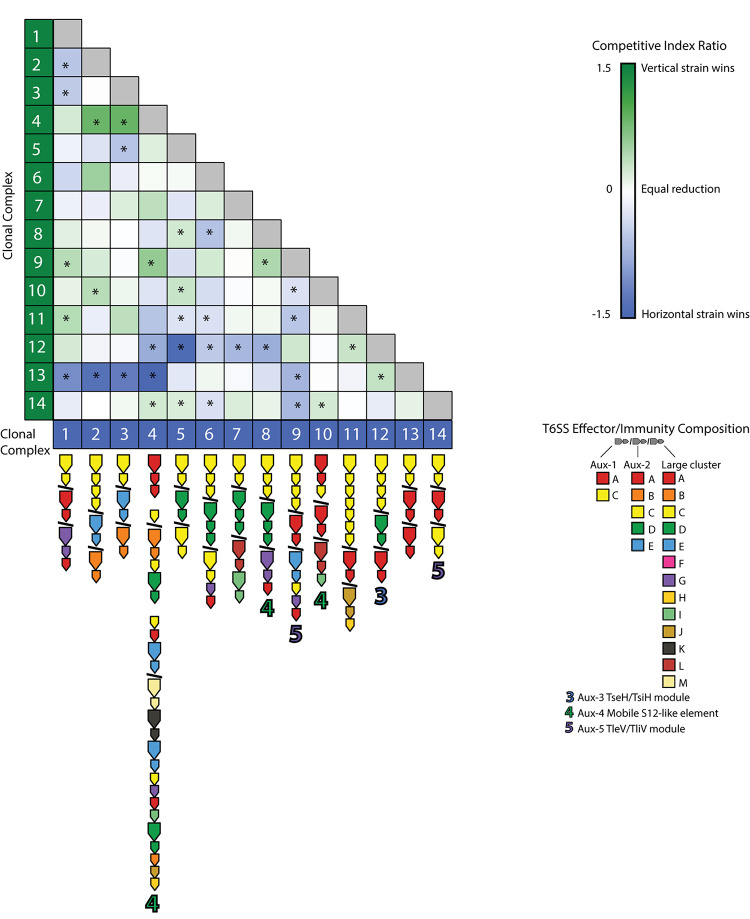
Pairwise competition of members from an environmental *Vibrio cholerae* population. Strains were competed in equal concentration at 25°C over 4 h. The mean log competitive index was visualized for all competitions, and color and intensity within each cell indicate a favorable outcome for the strain listed on the left (green) or bottom (blue). E-I module composition is indicated below strain names. Asterisks (*) indicate statistically significant results from a two-tailed, one-sample Student’s *t* test (*p* < 0.05).

**FIGURE 6:**
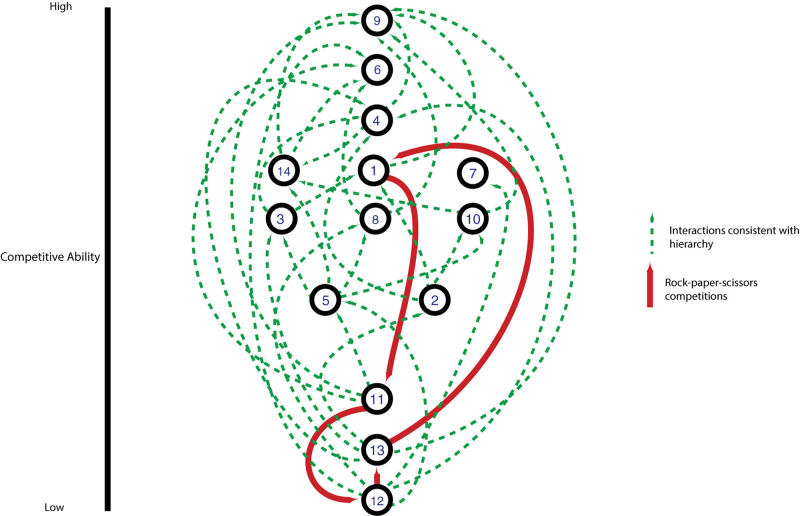
Competitive network of an environmental *Vibrio cholerae* population. Numbered nodes indicate individual strains; edges indicate pairings with statistically significant competitive outcomes. Strains are ranked from most to least competitive on the *y*-axis based on a scoring system where a statistically significant competitive outcome is worth two points and a draw is worth one point. Arrows point toward winning strain in an individual competition, with green arrows indicating outcomes upward or level in the hierarchy and red arrows highlighting a lower strain winning against a higher-ranked strain.

Most pairwise interactions between *V. cholerae* strains, both in this experiment and presumably in nature, occur between bacteria sharing few or no E-I modules. The majority of such pairings between incompatible strains resulted in even matchups, indicating that most of the population is able to weather incoming attacks of co-occurring strains (Figure 5). In around a quarter of competitions, however, one strain outcompeted the other (Figure 5). To understand if these successes are the results of some combinations of effectors and immunity proteins being more efficient than others, we visualized the competitive network of Oyster Pond strains using a simple (and arbitrary) scoring scheme (Figure 6). For this, we ranked strains vertically by competitive ability, which was calculated by awarding two points for significantly outcompeting another strain (*p* < 0.05) and one point for a stalemate. Using that scheme, CC9, which outcompetes seven of 13 strains and is not outcompeted by any of them, is placed squarely at the top of the hierarchy, while CC12, which is beaten by six strains and only outcompetes a single strain, is at the bottom. While higher-ranked strains generally outcompete lower-ranked ones, we observed an extended rock–paper–scissors interaction involving several strains—CC1 < CC11 < CC12 < CC13 < CC1. Additionally, the three most successful strains (CC9, CC6, and CC4) all encode the C-, G-, and A-type immunity genes in their large locus, and their success might be explainable by the observation that 50% of all strains in the dataset contain an effector neutralized by these immunity proteins (Figure 1). Thus, whether a strain is competitive or not depends very much on the composition of the population as a whole and the E-I module diversity found inside it.

## Discussion

From the genomic and experimental investigation of T6SS-mediated competition between members of an environmental *V. cholerae* population, we find that despite the large diversity of E-I modules encoded in distantly related genomes, most strains that would meet each other in nature are in fact equally matched. This has important ecological implications regarding the structure of such populations. Even strains with comparatively weak EI module combinations such as CC7 or CC3 are capable of stalemating more than half of the strains in this population. This is especially relevant when considering that in natural situations, two strains would rarely meet in equal concentrations, diminishing the effect a potentially superior E-I module composition could have. Similar interactions have been observed among co-occurring bacteria in *Streptomyces*, where the survival of potentially conflicting groups is posited to allow diversification of metabolic pathways and substrates utilized in a complex microbial community (Wright and Vetsigian, 2016; McNally et al., 2017; Steinbach et al., 2020). The T6SS has previously been demonstrated to maintain distinct bacterial groups through spatial segregation, with antagonism occurring at the edge of incompatible groups (Wong et al., 2016; McNally et al., 2017; Yanni et al., 2019; Steinbach et al., 2020). Our results thus support a model where the diversity of *V. cholerae* populations is maintained through the creation of a patchwork of microniches by T6SS-mediated competitive exclusion. Coexistence of incompatible strains can thus be maintained over long periods of time when ecological processes such as interactions and dispersal are spatially limited (Kerr et al., 2002). We anticipate the T6SS to be involved with regulating population dynamics on a microcosm level, as environmental bacteria colonize surfaces such as detritus, hosts, and heterogeneous particulate matter (Grossart et al., 2003). New particles are formed consistently in natural habitats due to turnover of organic material, and here we tested the competitive ability of strains to establish themselves as primary colonizers and maintain their occupation of space by contact-dependent competition. It is reasonable to expect that antagonism due to T6SS interactions will also influence population shifts observed in the successional colonization of these particles, with more fit genotypes outcompeting weaker primary colonizers (Dang and Lovell, 2016).

We also find evidence that HGT-mediated diversity of T6SS E-I modules can profoundly influence the competitive dynamics in a bacterial population. Given an otherwise identical E-I module composition, orphan immunity genes—remnants of past HGT events that replaced an ancestral effector gene with a new E-I module—appear to confer a predictable, positive effect to the recipient. Similarly, HGT may contribute to reduce competition between strains by homogenizing E-I module content. For example, the shared environmental success of CC3 and CC2, which are both widely abundant in Oyster Pond, could be the result of a past confrontation, leading to one strain replacing its E-I module with that of another to create a temporary truce between former competitors. Reduced competition between distantly related strains with compatible E-I modules could provide a mutual advantage in situations where more than two bacterial strains are present, their compatibility allowing them to outcompete others. However, given the dynamic nature of E-I module composition, it stands to reason that the rough hierarchy established in this work is very short lived—while the incorporation of new E-I modules through DNA uptake of lysed neighbors may refine and improve upon the repertoires of less fit strains, it may also cause higher-performing strains to exchange stronger E-I with weaker replacements at their detriment (Borgeaud et al., 2015; Thomas et al., 2017). In particular, strains with highly competitive E-I module combinations are more likely to take up DNA from the lysis of defeated weaker ones. As the replacement of an E-I module often leads to the retention of an immunity gene, and this immunity gene in combination with a new effector could provide a considerable competitive advantage against strains with otherwise similar E-I modules, this downgrade in E-I efficacy would be momentarily selected for. Thus, a previously dominant strain could quickly find itself with a suboptimal E-I module composition, leading to the rise of different strains. Additionally, the possession of nominally weaker E-I module combinations could, under certain situations, be beneficial due to density-dependent fitness effects. For example, in situations where CC1 becomes more abundant, the weak strain CC11 should be at an advantage due to its ability to specifically outcompete that strain. As strain composition in *V. cholerae* populations have been shown to be highly dynamic (Kirchberger et al., 2020), such scenarios do not appear unlikely.

It is tempting to conclude that the outcome of T6SS competition between distantly related strains could be reliably predicted by their E-I module composition alone (without taking into account the rest of the organism). However, our results and a number of other studies have also demonstrated the limits of such predictions. Recently, Drebes Dörr and Blokesch demonstrated that *V. cholerae* strains collected from different locations in California are still capable of killing each other despite identical E-I module composition (Drebes Dorr and Blokesch, 2020). Furthermore, Troselj et al. (2018) showed that unequal gene expression between strains can lead to the suboptimal production of immunity proteins, which are then unable to abrogate the toxicity of injected effectors. In our study, CC4, a strain that encodes a plethora of immunity genes and therefore should be protected from any competitor, is in fact not. In its case, the complex structure of its T6SS loci makes it unclear whether particular genes are part of an actively expressed operon and thus even have a phenotypic effect, a reasonable assumption for strains with shorter and uninterrupted T6SS gene arrays. Environmental factors also greatly influence the outcome of competitions. For example, it has previously been hypothesized that the superior competitive ability of the pathogenic strain V52 is due to the highly conserved and lethal E-I module combination within the PG lineage. However, from our results, it appears that at least in the aquatic reservoir of cholera, environmental *V. cholerae* may be better competitors at lower temperatures compared to temperatures found in the human body, under which T6SS competition assays have typically been performed (MacIntyre et al., 2010; Unterweger et al., 2012, 2014; Crisan et al., 2019; Drebes Dorr and Blokesch, 2020). Whether this temperature dependence of outcomes is a matter of different growth rates or more complex phenotypic changes remains to be determined. Numerous other factors and environmental variables could have complex effects on T6SS-mediated competition. For example, biofilms have been shown to act as a protective layer around *V. cholerae* to shield cells from exogenous T6SS attacks (Toska et al., 2018). Furthermore, growth rate and motility of competing strains have been identified as factors important in displacing competitors (Gude et al., 2020). The presence of envelope stress protein has also been shown to confer immunity to certain T6SS effectors (Hersch et al., 2020). On the other hand, the presence or absence of certain genes (such as the caseinolytic protease genes *clpP* and *clpA*, or the disulfide bond formation gene *dsbA*) can also increase a strain’s susceptibility to T6SS attacks (Lin et al., 2019; Mariano et al., 2019). Even the simple addition of glucose to the growth medium can have drastic effects on competition (Crisan et al., 2021). As such, although an important component, E-I module composition alone is obviously not the sole deciding factor determining the outcome of T6SS-mediated competition, or competition as a whole. Future studies into ecologically relevant competition assays may also consider adaptations to media compositions to reflect aquatic environmental conditions that are generally more oligotrophic (Lambert et al., 2019).

Given the role of T6SS in stalemating bacterial competitors, environmental, ecologically differentiated non-pathogenic strains of *V. cholerae* such as the ones studied in this work could play an important role in preventing the spread of pandemic *V. cholerae* internationally. Global ocean currents and ship ballasts are likely to have spread occasional pandemic *V. cholerae* bacteria across the globe (Lipp et al., 2002; Ramamurthy et al., 2019; Deen et al., 2020) but local *V. cholerae* (Almagro-Moreno and Taylor, 2013; Sakib et al., 2018) might have prevented them from becoming a permanent part of the immediate flora. Only when overwhelming concentrations of pandemic *V. cholerae* are introduced *via* a human vector, numbering in the trillions released by a cholera victim (Almagro-Moreno et al., 2015), might they be able to gain a foothold against a diverse and locally adapted pre-existing community of *V. cholerae*. In such cases, competition still continues in reservoirs and may have downstream effects for invaders: Haiti, for example, has had no new cholera cases for over a year (UN News, 2020), and this may be due in part to the extant environmental community successfully antagonizing pandemic *V. cholerae*.

## Materials and Methods

### Strain Selection and Growth

All environmental strains of *V. cholerae* used originated from Oyster Pond, MA, United States, with isolation protocols as previously described (Kirchberger et al., 2016). The clinical isolate *V. cholerae* V52 was chosen to represent PG cholera strains expressing a constitutively active T6SS. *E. coli* K12 substrain MG1655 was used in control experiments to ensure that T6SS was actively expressed. Spontaneous rifampicin-resistant mutants were generated by recovering mutant spread plated on rifampicin-supplemented Luria-Bertani (LB) (Difco) plates as performed in MacIntyre et al. (2010).

### E-I Module Typing

To identify conserved chromosomal T6SS loci, genes downstream of aux-1, aux-2, and the large cluster E-I modules (VC1421, VCA0022, and VCA0125 of *V. cholerae* strain N16961 respectively), were mapped to each environmental *V. cholerae* genome in Geneious 6.1.8. Putative immunity genes were extracted and classified based on previous reference sequences for each E-I family as previously described (Unterweger et al., 2014), followed by effectors for each locus. The presence of aux-5 was queried through the T6SS predictor pipeline (Crisan et al., 2019) in addition to Geneious mapping.

### Phylogenetic Tree Construction

Genomes for all isolates have been sequenced as previously described (Kirchberger et al., 2016). Whole genome alignment of strains was performed using mugsy version (Angiuoli and Salzberg, 2011) and gaps were removed, resulting in an alignment of 2,948,969 bp. A whole genome phylogeny was subsequently built using the GTR + GAMMA substitution model implemented in RAxML (Stamatakis, 2014), with branch support assessed with 100 fast-bootstrap pseudoreplicates. Individual alignments of effector and immunity genes from Oyster Pond strains and reference strains from a previous publication (Kirchberger et al., 2017) were aligned using ClustalOmega (Sievers and Higgins, 2014) and standard settings, and phylogenetic trees were constructed as above.

### Competition Assay

Competition assay protocols were adapted from previously described methods (MacIntyre et al., 2010). Overnight cultures of *V. cholerae* or *E. coli* were grown at 37°C on LB agar supplemented with rifampicin when appropriate. Cells were harvested, and the concentration of strains was normalized by OD_600_ to 10^7^ cfu/ml. Rifampicin-resistant and rifampicin-sensitive WT strains were resuspended in LB broth in 1:1 ratios of 10^7^ cfu/ml, and 25 μl of the mixture was spotted on prewarmed LB agar plates and incubated at either 25°C or 37°C for 4 h. For each replicate, 25 μl of the starting mixture was suspended in 975 μl of LB, serially diluted and spot plated onto LB or LB with rifampicin (LB + R) to determine starting concentrations of each strain. After incubation, each spot was harvested completely and resuspended in 1 ml of LB, which was then serially diluted 10-fold to 10^–7^ concentration. A 10 μl aliquot from each dilution was spot plated on both LB and LB + R plates in duplicate. Each pairwise experiment was performed with six replicates. Plates were incubated at 37°C, and colony-forming units (CFUs) were counted at the highest dilution recovered. Single CFUs were counted if the previous dilution was accurately observed to be 10-fold greater, i.e., approximately 10 CFUs were found before the single CFU. Concentration of the WT strain was determined by subtracting CFUs on LB + R from CFUs enumerated on LB. Control competition assays were performed to ensure all *V. cholerae* T6SS were functioning as expected by competing strains against *E. coli* K12 substrain MG1655, an isolate that lacks a T6SS (Supplementary Figure 3).

### CI and Survivor Percentage Calculation

Competitive indices were determined by comparing concentrations of survivors to starting cultures as performed previously (Unterweger et al., 2014). CFUs/ml of rifampicin-resistant strains were divided by CFUs/ml of WT, for both starting and recovered time points. The ratio after 4 h was divided by the starting concentration to calculate CI. The average of surviving strains was calculated by dividing the CFUs at 4 h over the starting concentration per strain and converted into a percentage.

### Supernatant Assay

Cell-free supernatant was harvested from overnight cultures grown as described above through serial centrifugation at 13,000 rpm for two 10-min intervals. Target strains were spread on LB + R plates, and 2 μl supernatant aliquots were spotted on the dried spread plates. Plates were incubated overnight at 37°C, and plaque formation was visually inspected the following day.

### Statistics and Visualization

A one-sample Student’s *t* test determined statistical significance of CI values against an assumed comparative mean of 0. For Figure 3B, a one-factor ANOVA was performed to assess statistical significance of mean reduction of strains based on predicted interaction, in addition to a two-sample unpaired *t* test. All statistical analyses were preceded by tests of normality on the data. Dot plots were generated in R using ggplot2 (Wickham, 2016; R Core Team, 2019). The competitive hierarchy graph was generated in Cytoscape (Shannon et al., 2003). All figures were processed afterward in Adobe Illustrator CS6.

## Data Availability Statement

The raw data supporting the conclusions of this article will be made available by the authors, without undue reservation.

## Author Contributions

NH and PK contributed equally to this research: author order was decided alphabetically by surname. NH performed all bench work assays, analyzed experimental data, and generated competition figures. PK analyzed *Vibrio cholerae* genomes and constructed phylogenetic trees. NH and PK wrote the manuscript. NH, PK, RC, and YB were involved with manuscript editing. The study was supervised and funded through the labs of RC and YB. All authors were involved in the creation and optimization of the experimental design and had input on how best to analyze raw data.

## Conflict of Interest

The authors declare that the research was conducted in the absence of any commercial or financial relationships that could be construed as a potential conflict of interest.
