# Supplementary material for: Modular Molecular Weaponry Plays a Key Role in Competition Within an Environmental Vibrio cholerae Population
Source: Front Microbiol. 2021 May 21;12:671092. doi: 10.3389/fmicb.2021.671092 (PMC8189183; doi:10.3389/fmicb.2021.671092)
Supplement: Supplementary file 1 [file Table_1.docx]

| Species | Strain | CC* | *viuB* | T6SS Effector Immunity | Accession Number | Year + | References |
| --- | --- | --- | --- | --- | --- | --- | --- |
|  |  |  | allele |  |  | Source Isolated |  |
| *Vibrio cholerae* | OYP6F10 | 1 | *viuB-39* | C/A/Ga | NMSZ00000000 | 2009 | Orata et al. (2015) |
|  |  |  |  |  |  | Oyster Pond, MA, USA |  |
| *Vibrio cholerae* | OYP2E01 | 2 | *viuB-64* | Cccc/E/B | NMTK00000000 | 2009 | Orata et al. (2015) |
|  |  |  |  |  |  | Oyster Pond, MA, USA |  |
| *Vibrio cholerae* | OYP4G06 | 3 | *viuB-30* | C/E/B | LBGH00000000 | 2009 | Orata et al. (2015) |
|  |  |  |  |  |  | Oyster Pond, MA, USA |  |
| *Vibrio cholerae* | OYP2A12 | 4 | *viuB-43* | Aaaa-c/BcD-caE/MKEcgaiDbjh/4 | NMTN00000000 | 2009 | Orata et al. (2015) |
|  |  |  |  |  |  | Oyster Pond, MA, USA |  |
| *Vibrio cholerae* | OYP4B01 | 5 | *viuB-41* | C/D/C | NMTI00000000 | 2009 | Orata et al. (2015) |
|  |  |  |  |  |  | Oyster Pond, MA, USA |  |
| *Vibrio cholerae* | OYP7G04 | 6 | *viuB-61a* | Ccc/Ddd/Cga | QEAX00000000 | 2009 | Orata et al. (2015) |
|  |  |  |  |  |  | Oyster Pond, MA, USA |  |
| *Vibrio cholerae* | OYP6E10 | 7 | *viuB-60* | C/Ddd/LI | QEBI00000000 | 2009 | Orata et al. (2015) |
|  |  |  |  |  |  | Oyster Pond, MA, USA |  |
| *Vibrio cholerae* | OYP6D09 | 8 | *viuB-61b* | Ccc/Ddd/Ga4 | QEBM00000000 | 2009 | Orata et al. (2015) |
|  |  |  |  |  |  | Oyster Pond, MA, USA |  |
| *Vibrio cholerae* | OYP2D07 | 9 | *viuB-62a* | Cccc/A/Ecga/5 | NMTL00000000 | 2009 | Orata et al. (2015) |
|  |  |  |  |  |  | Oyster Pond, MA, USA |  |
| *Vibrio cholerae* | OYP1G01 | 10 | *viuB-29* | Ac/A/Li/4 | NMTO00000000 | 2009 | Orata et al. (2015) |
|  |  |  |  |  |  | Oyster Pond, MA, USA |  |
| *Vibrio cholerae* | OYP4H04 | 11 | *viuB-49* | Ccccccc/A/Jh | QEBY00000000 | 2009 | Orata et al. (2015) |
|  |  |  |  |  |  | Oyster Pond, MA, USA |  |
| *Vibrio cholerae* | OYP4D04 | 12 | *viuB-62b* | Cccc/D/A | QECE00000000 | 2009 | Orata et al. (2015) |
|  |  |  |  |  |  | Oyster Pond, MA, USA |  |
| *Vibrio cholerae* | OYP4C07 | 13 | *viuB-34* | C/A/A | LBGE00000000 | 2009 | Orata et al. (2015) |
|  |  |  |  |  |  | Oyster Pond, MA, USA |  |
| *Vibrio cholerae* | OYP4E08 | 14 | *viuB-53* | C/A/C/5 | QECB00000000 | 2009 | Orata et al. (2015) |
|  |  |  |  |  |  | Oyster Pond, MA, USA |  |
| *Vibrio cholerae* | V52 |  | *viuB-73* | Ac/A/A | AAKJ00000000 | 1968 | Chun et al. (2009) |
|  |  |  |  |  |  | Sudan (Clinical) |  |
| *Escherichia coli* | K12 substr. MG1655 |  |  |  | U00096 | 1997 | Blattner et al. (1997) |
|  |  |  |  |  |  | Wild-type lab strain |  |

**Supplementary Table 1. List of all isolates used in competition assays and effector-immunity (EI) combinations of aux-1, aux-2 and large cluster.** Blank cells indicate no relevant metadata. Clonal complex (CC), *viuB*, and EI typing schemes listed in literature cited list. Slashes designate loci positions. Dashes denote gaps between genes. Uppercase letters indicate effector-immunity; lower case letters are orphan immunity genes. Numbers at the end of EI specify aux3-5 presence.

Literature Cited

Blattner FR, *et al*. (1997). The complete genome sequence of *Escherichia coli* K-12. Sci. DOI:10.1126/science.277.5331.1453

Chun J, *et al*. 2009. Comparative genomics reveals mechanism for short-term and long-term clonal transitions in pandemic *Vibrio cholerae*. Proc Natl Acad Sci USA. DOI:106:15442-15447.

Kirchberger PC, *et al*. 2016. A small number of phylogenetically distinct clonal complexes dominate a coastal *Vibrio cholerae* population. Appl Environ Microbiol. DOI:10.1128/AEM.01177-16

Kirchberger PC, *et al*. 2017. Sequential displacement of Type VI secretion system effector genes leads to evolution of diverse immunity gene arrays in *Vibrio cholerae*. Sci Rep. DOI:10.1038/srep45133

Kirchberger PC, *et al*. 2020. Culture-independent tracking of *Vibrio cholerae* lineages reveals complex spatiotemporal dynamics in a natural population. Environ Microbiol. DOI:10.1111/1462-2920.14921

Orata FD, *et al*. 2015. The dynamics of genetic interactions between *Vibrio metoecus* and *Vibrio cholerae*, two close relatives co-occurring in the environment. Genome Biol Evol. DOI:10.1093/gbe/evv193.

Unterweger, D., *et al*. 2014. "The *Vibrio cholerae* type VI secretion system employs diverse effector modules for intraspecific competition." Nat Commun. DOI: 10.1038/ncomms4549
